# The Combination of a Graph Neural Network Technique and Brain Imaging to Diagnose Neurological Disorders: A Review and Outlook

**DOI:** 10.3390/brainsci13101462

**Published:** 2023-10-16

**Authors:** Shuoyan Zhang, Jiacheng Yang, Ying Zhang, Jiayi Zhong, Wenjing Hu, Chenyang Li, Jiehui Jiang

**Affiliations:** 1 School of Communication and Information Engineering, Shanghai University, Shanghai 200444, China; 2 School of Life Sciences, Shanghai University, Shanghai 200444, China; 3 Shanghai Institute of Biomedical Engineering, Shanghai University, Shanghai 200444, China

**Keywords:** neurological disorder, deep learning, graph neural network, diagnostic model

## Abstract

Neurological disorders (NDs), such as Alzheimer’s disease, have been a threat to human health all over the world. It is of great importance to diagnose ND through combining artificial intelligence technology and brain imaging. A graph neural network (GNN) can model and analyze the brain, imaging from morphology, anatomical structure, function features, and other aspects, thus becoming one of the best deep learning models in the diagnosis of ND. Some researchers have investigated the application of GNN in the medical field, but the scope is broad, and its application to NDs is less frequent and not detailed enough. This review focuses on the research progress of GNNs in the diagnosis of ND. Firstly, we systematically investigated the GNN framework of ND, including graph construction, graph convolution, graph pooling, and graph prediction. Secondly, we investigated common NDs using the GNN diagnostic model in terms of data modality, number of subjects, and diagnostic accuracy. Thirdly, we discussed some research challenges and future research directions. The results of this review may be a valuable contribution to the ongoing intersection of artificial intelligence technology and brain imaging.

## 1. Introduction

NDs, including Alzheimer’s disease, Parkinson’s disease, etc., are the leading cause of disability and the second leading cause of death in humans [1,2,3]. It is important to explore the disease mechanism and diagnose NDs at an early stage. Currently, various imaging techniques are used to peer inside the brain, such as magnetic resonance imaging (MRI), electroencephalogram (EEG), and positron emission computed tomography (PET). Particularly, artificial intelligence technology combined with neuroimaging has been widely used because of its high classification accuracy [4]. For example, the large model known as GPT [5] has broken through the technical boundaries of artificial intelligence, and has brought changes to many application fields. In the medical field, many researchers are beginning to apply large models for ND diagnosis, prevention, and treatment [6]. Convolutional Neural Network (CNN) [7] and Long Short Term Memory (LSTM) [8] have been adopted in many ND studies because of their good capability at extracting the spatial and temporal features of the brain [9,10]. However, NDs result in alterations in brain functional and structural connections, as well as local and global connections [11,12], and traditional deep learning models such as CNN and LSTM are difficult to fit to the connectivity of the brain. Therefore, researchers have modelled human brains using graph methods to extract abnormal brain networks, subnetworks, and local connections [13,14,15].

**Figure 1:**
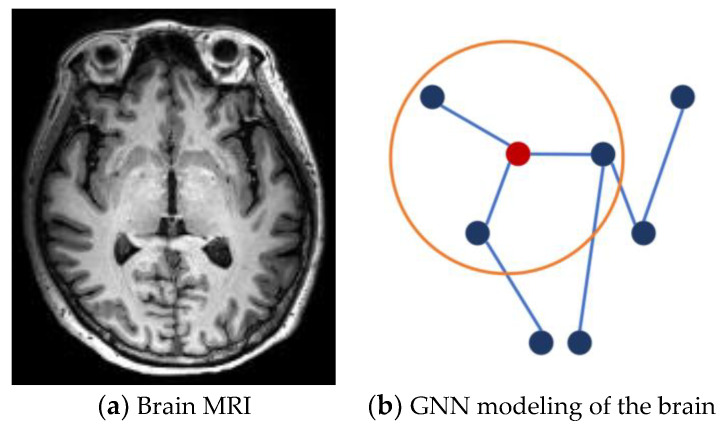
The schematic diagram of GNN modeling for the brain. (**a**) Brain MRI: a slice of the brain T1-MRI. (**b**) GNN modeling of the brain: Nodes represent brain regions and edges represent connections between brain regions. The connectivity features are extracted by the relation of adjacent nodes. Inside the ring is the central node and its first-order neighbors.

A GNN combines the advantages of graph and deep learning [16]. In the analysis of GNN models, the brain is divided into several regions. Each brain region can be represented by a node, and the connectivity between two nodes can be represented by an edge [17,18]. By means of spectral convolution or spatial convolution, GNN models aggregate and transform the features of adjacent nodes on the graph to extract topological information. During this process, abnormal brain region and connectivity will be extracted. A GNN model of the brain is shown in Figure 1. For example, T1 weighted imaging (T1-MRI) can be constructed as a graph of the spatial relationships of brain regions. The GNN is then calculated on the constructed brain network.

Due to the superiority of the GNN, researchers have investigated GNNs in the field of medical health. Ahmedt-Aristizabal et al. [4] widely investigated the application of GNNs in disease diagnosis. Bessadok et al. [19] investigated GNNs in neuroscience from the three dimensions of domain, resolution, and time. Although these investigations provide comprehensive information, they are not detailed enough on how GNN is used in the diagnosis of NDs. Our aim is to provide a more detailed survey of the techniques and applications to help readers quickly understand and get started in this area of research. Therefore, this review focuses on the combination of a GNN with brain imaging and their application in the diagnosis of NDs. The scientific contributions of this paper include the following:

(1) This paper systematically investigated the technological framework of a GNN and discussed the advantages and disadvantages of different GNN models for different neuroimaging signals.
(2) This paper investigated the applications of different GNN models in a variety of NDs, such as Alzheimer’s disease [20], Parkinson’s disease [21]., etc. This may indicate the potential clinical values of GNN models.

The rest of this review is organized as follows. In Section 2, the computational framework of the GNN is introduced. In Section 3, the applications of GNNs in a variety of NDs are investigated. In Section 4, we present some research shortages and challenges, and summarize future research directions. Finally, we summarize the advances of GNNs combined with brain imaging in the diagnosis of NDs in Section 5.

## 2. Framework of a Graph Neural Network for NDs

**Figure 2:**
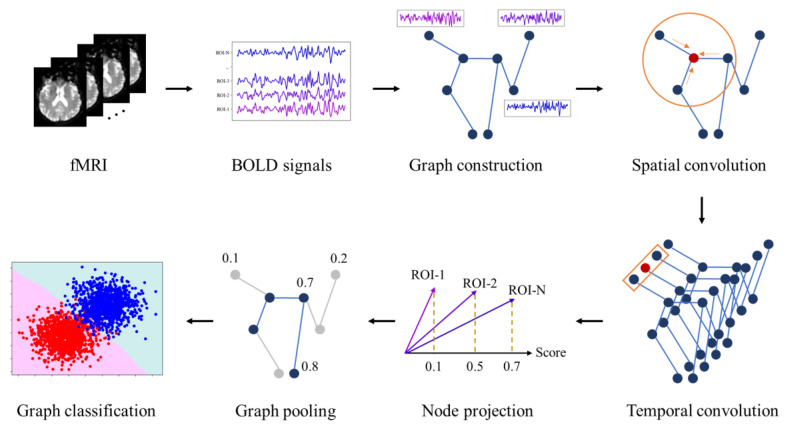
Framework of GNN for ND. The entire framework begins by extracting the BOLD signal from fMRI. Next, Pearson correlation is used to construct graph. Subsequently, spatial convolution and temporal convolution are applied to extracted spatiotemporal features. Node weights are obtained through node projection. Finally, graph pooling is employed to achieve graph embedding representation, which is then used for classification.

In this section, we systematically investigated each computing module of a GNN in the diagnosis of ND. This includes graph construction, graph convolution, graph pooling, and graph prediction. We would like to provide a detailed overview of GNN technology in this field. The framework of the GNN for ND is shown in Figure 2. Taking functional MRI (fMRI) as an example, the blood oxygen level-dependent (BOLD) signals are first extracted from the fMRI, and then the graph is constructed for GNN calculation. Spatial convolution and temporal convolution are used to extract spatiotemporal features. Node projection and graph pooling implement information filtering. Finally, diagnosis is realized through graph classification.

In order to further understand the diagnostic application of GNN in NDs, we briefly introduce basic knowledge on GNNs. A graph can be represented by G=(V, E), where V denotes a set of nodes and E denotes a set of edges. Nodes may have attributes, represented by $X^{V}\in R^{|V|\times d}$, and edges may also have attributes, represented by $X^{E}\in R^{|E|\times b}$. |V| denotes the node number and |E| denotes the edge number. d and b are the feature dimensions of the node attributes and edge attributes, respectively. A node is represented as $v_{i}$, and an edge between two nodes is represented as $e_{ij}=(v_{i},v_{j})$. An adjacent node set is denoted as $N\left(v\right)=\{u\in V|(v,u)\in E\}$. Sometimes, the adjacency relationship is represented by an adjacency matrix $A\in R^{\left|V|\times |V\right|}$ [22].

**Figure 3:**
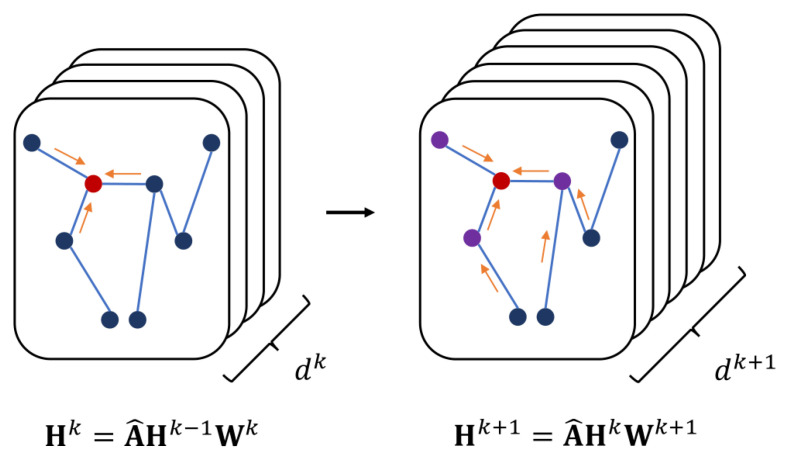
The calculation process of GNN. The hidden features were multiplied by the normalized adjacency matrix and learnable parameters to obtain the hidden features of the next layer. At the same time, the dimension of the hidden features also changed. The hidden features were equivalent to the feature maps in CNN.

A GNN is neural model that captures the dependence relationship of topology via message-passing between the nodes of graphs [16]. Therefore, W is used to represent the learnable parameters of GNN, H denotes the hidden features obtained via GNN calculation, and $h_{v}$ represents the hidden features of node v. The activation function is σ(·). k denotes the index of the layer. The calculation process of GNN is shown in Figure 3.

### 2.1. Graph Construction

Before applying the GNN, it is essential to organize the data into graphs. The form of the graphs can be categorized into two types: population graphs and subject graphs. From a macro perspective, the population graph treats each subject as a node, with demographic information and feature similarities between subjects serving as the edges. From a micro perspective, the subject graph divides the brain into multiple regions. Each region acts as a node, and the functional and structural information between brain regions is utilized to establish the edges.

In the construction graph method, there are the Pearson correlation coefficient, partial correlation coefficient, Euclidean distance, and attention mechanism. Table 1 summarizes the common methods used to construct the graph.

#### 2.1.1. Population Graph

To describe the relationship between subjects, image (T1-MRI, fMRI, etc.) and non-image information (age, gender, gene, etc.) are often used to construct the graph.

Rakhimberdina et al. [23] used the hamming distances of age, gender, acquisition site to construct a population graph. Jiang et al. [30] took functional connection from fMRI as the node feature and used a Gaussian kernel to compute edges between nodes. Parisot et al. [24] integrated image features with non-image data. They calculated an adjacency matrix for image features (functional connection, brain volume) using a Gaussian kernel, and another adjacency matrix was computed for non-image information (age, gender, acquisition site, etc.) using a thresholding method. These two adjacency matrices were then combined through the Hadamard product to create the final adjacency matrix. In studies [21,25,26,27,28,31,32], researchers have also used the same method to construct population graphs.

Some studies construct edges based on the cosine similarity of node features. In their study, Huang et al. [33] utilized image data for extracting node features and non-image data for constructing edges. They derived edge weights from the non-image data through the use of a Multilayer Perceptron (MLP) and cosine similarity. Zheng et al. [34] multiplied the node features with the parameter matrix, and then constructed the edge between subjects using cosine similarity. Lin et al. [35] employed an encoder to extract site-invariant information and site-specific information from fMRI data. Subsequently, they utilized the site-specific information and phenotypic data to construct a population graph using the cosine similarity function. Pan et al. [36] constructed two population graphs based on functional image features and phenotypic features, respectively. The functional graph was constructed using cosine similarity and K-nearest neighbors (KNN), and the phenotypic graph was constructed adaptively using a pair association encoder [33].

In addition, Song et al. [37] employed an attention mechanism to integrate the node features, gender, device information, multicenter information, and disease status of the training set samples to construct a multi-center attention graph.

#### 2.1.2. Subject Graph

In the subject graph, the brain template is used to divide the brain into regions, with brain regions as nodes and functional and structural relationships between brain regions as edges.

Pearson correlation and partial correlation are the most used methods of constructing graphs. Zhao et al. [46] utilized Pearson correlation to create the adjacency matrix and adopted partial correlation as the node feature. Nevertheless, the process of constructing graphs inevitably introduces some noise. These unwanted noises can be effectively filtered out through threshold processing. In works [39,40], they constructed the graph using Pearson correlation and retained the positive coefficients as edges. Wang et al. [41] adopted a Pearson correlation construction graph and took the correlation coefficient greater than 0.4 as the connection. In works [42,43,44,45], they established the graph using Pearson correlation and then binarized the edge weights through a thresholding process. Li et al. [61] constructed their graph using the partial correlation of BOLD signals, and took the top 10% of positive correlations as edges to ensure that there were no isolated nodes in the graph.

Some studies employ the constructing of graphs as a method for tuning hyperparameters. Klepl et al. [65] selected eight methods for constructing functional connectivity from EEGs, including the absolute value of Pearson correlation, mutual information, etc. Shan et al. [66] applied six methods to construct a graph, which were Pearson correlation, magnitude-squared coherence, imaginary part of coherence, wavelet coherence, phase locking value, and the phase lag index. Chang et al. [63] used the partial correlation coefficient and phase lag index. Li et al. [64] calculated the Pearson correlation, partial correlation, and geometric distance of the Region of Interest (ROI) as edges.

Given that node features are dynamic signals that change over time, several studies have explored the extraction of temporal features for the construction of graphs. Yang et al. [67] used a Gated Recurrent Unit (GRU) [70] to extracted node features from both the functional and structural network. They further constructed an adaptive adjacency matrix based on the inner product of these node features. Lee et al. [38] extracted features from BOLD signals in the brain region through CNN, and then selected the important nodes through reinforcement learning. The correlation distance calculated the edge weights between important nodes according to features. Likewise, Mahmood et al. [69] employed CNN to extract the features from the BOLD signal, and then constructed directed, weighted, functional connectivity using a multi-head self-attention mechanism.

Various construction methods encompass complementary information, prompting some studies to simultaneously utilize multiple graphs. Yao et al. [57] employed four templates, ranging from coarse to fine, to partition brain regions and constructed brain networks using Pearson correlation and KNN. He et al. [71] extracted the human skeleton from a video and proceeded to create a local information graph based on the natural connections between joints. Following this, they designated the neck joint as the central point and connected other nodes to it to establish a global information graph. Furthermore, apart from constructing multigraphs in spatial dimensions, it is also feasible to create multigraphs from time series. Wang et al. [58] divided fMRI into multiple sub-sequences along the time axis. Pearson correlation was used in each sub-sequence, and the dynamic functional network was obtained according to proportional threshold.

Most of the studies mentioned above are conducted within the context of homogeneous graphs. However, in different scenarios, the nodes and edges within the graph can belong to different types. Yao et al. [60] established heterogeneous graphs comprising two types of nodes: functional nodes and structural nodes. They employed Pearson correlation to create edges between functional nodes, fractional anisotropy for the edges between structural nodes, and physical relationships for the edges connecting functional and structural nodes.

Across various MRI techniques, it is known that fMRI reveals functional connections, while diffusion MRI (dMRI) reveals structural connections. Consequently, some researchers choose to construct graphs using fiber tracking algorithms grounded in Diffusion Tensor Imaging (DTI). Huang et al. [72] used a deterministic tracking algorithm to calculate DTI fiber bundles, and took the 10 nearest neighbor nodes to construct the graph. Liu et al. [73] selected the features of DTI and reconstructed the topology of the structural MRI (sMRI), and combined it with the Pearson correlation coefficient of fMRI to construct brain connectivity. Subaramya et al. [74] used fiber bundles and brain regions’ volumes to construct a weighted graph, and then obtained a binarized graph through the sign test.

### 2.2. Graph Convolution

Once the graph is constructed, features can be extracted through graph convolution. Graph convolution leverages the graph’s topology to facilitate message-passing between nodes, enabling the extraction of high-level and abstract features. Graph convolution can be applied to both population and subject graphs. The GNN diagnostic model for NDs typically includes fundamental graph convolution techniques, which we will briefly introduce here.

**ChebNet**. Since the graph convolution kernel of the spectral network [75] is global and computationally complex, Defferrard et al. [76] used the Chebyshev polynomial approximation to calculate graph convolution. The calculation method is shown in Equation (1).

(1) $$g\;★\;x\;\approx \;\sum_{k=0}^{K}w_{k}T_{k}\left(\tilde{L}\right)x$$

where $\tilde{L}=\frac{2}{\lambda_{max}}L-I_{N}$ is a matrix of scaled eigenvalues. $\lambda_{max}$ is the largest eigenvalue of L. $w_{k}$ is the coefficient of Chebyshev. Chebyshev polynomials can be denoted as $T_{k}\left(x\right)=2xT_{k-1}\left(x\right)-T_{k-2}\left(x\right)$, with $T_{0}\left(x\right)=1$ and $T_{1}\left(x\right)=x$.

**GCN**. Kipf et al. [77] simplified the Chebyshev graph convolution using the first order approximation. The operation can be written as Equation (2).

(2) $$H={\tilde{D}}^{-1/2}\tilde{A}{\tilde{D}}^{-1/2}XW$$

where $\tilde{A}$ is the normalized adjacency matrix, and $\tilde{D}$ is the degree matrix of $\tilde{A}$. X is the input node feature, and W is the learnable parameter matrix. Finally, the extracted hidden feature is denoted as H.

**GraphSAGE**. In order to adapt to the evolution of the graphs, Hamilton et al. [78] proposed an inductive learning framework of adjacent node sampling and aggregation. Sampling and aggregation are calculated as shown in Equation (3).

(3) $$\left\{\begin{array}{c} h_{N(v)}^{k}={agg}_{k}(\left\{h_{u}^{k-1},\forall u\in N\left(v\right)\right\})\\ h_{v}^{k}=\sigma (W^{k}\cdot concat(h_{v}^{k-1},h_{N(v)}^{k})) \end{array}\right.$$

${agg}_{k}$ denotes aggregation function, such as mean aggregator, pooling aggregator, etc.

**GAT**. Velickovic et al. [79] introduced the self-attention mechanism into GNN, where the weight of the edges is adaptively obtained through hidden features. The computing method is shown in Equation (4).

(4) $$\left\{\begin{array}{c} \alpha_{uv}=\frac{\exp(\sigma (a^{T}[Wh_{u}||Wh_{v}]))}{\sum_{p\in N(v)}\exp(\sigma (a^{T}[Wh_{p}|\left|Wh_{v}\right]))}\\ h_{v}^{k}=f(\sum_{u\in N(v)}\alpha_{uv}Wh_{u}^{k-1}) \end{array}\right.$$

where $\alpha_{uv}$ is the attention score, and f(·) represents the concatenating or averaging the multiple attention heads.

**GIN**. Inspired by the Weisfeiler-Lehman test, Xu et al. [80] proposed a graph isomorphism network and proved that its discriminant and representational ability is equal to the Weisfeiler-Lehman test. The calculation is shown in Equation (5).

(5) $$h_{v}^{k}={MLP}^{k}((1+\epsilon^{k})\cdot h_{v}^{k-1}+\sum_{u\in N(v)}h_{u}^{k-1})$$

where $\epsilon^{k}$ denotes a learnable parameter.

With these foundational graph convolutions, researchers can readily extract features from brain image data. In some studies, graph convolution serves as a layer within their models, enabling the extraction of spatial features between brain regions or electrodes [73,81]. In other studies, each brain region or electrode not only exhibits spatial correlation but also generates temporal signals, such as an EEG and fMRI. To capture this temporal dynamic information, researchers have introduced the spatial-temporal GNN [82,83]. Furthermore, various scales and distinct graph construction methods offer different perspectives for expressing graph information. Consequently, some studies employ multiple graphs simultaneously and propose the multi-graph GNN model [57,84]. In terms of feature extraction, these GNN models can be categorized into spatial feature extraction, spatial-temporal feature extraction, and multi-graph feature extraction.

A summary of commonly used graph convolutions in GNN models is provided in Table 2. For spatial feature extraction, we listed methods based on the graph convolution architecture above. In the context of spatial-temporal feature extraction, we included two prevalent methods: a recurrent neural network (RNN) [85] and CNN. The multi-graph feature extraction can be categorized into two parts: scale and construction methods. The former employs multiple templates to construct the graph, like AAL116 (Automated Anatomical Labelling with 116 ROIs) [86] and CC200 (Craddock with 200 ROIs) [87]. The latter involves utilizing various construction methods, such as Pearson correlation and mutual information.

#### 2.2.1. Spatial Feature Extraction

##### ChebNet-Based

ChebNet [76] is the earliest GNN model widely used by researchers. Numerous studies have built upon ChebNet to enhance its capabilities and apply it to the diagnosis of NDs. In the context of population graphs, Parisot et al. [24] and Liu et al. [26] extracted the image features from subjects as node features, and applied the Chebyshev graph convolution on the population graph to predict disease in a semi-supervised manner. For subject graphs, Liu et al. [73] and Qin et al. [55] utilized the Pearson correlation matrix as node features, and employed two Chebyshev graph convolutions followed by a fully connected layer to predict NDs.

##### GCN-Based

A GCN [77] further simplifies the calculation process of ChebNet and is the most used model in the diagnosis of NDs. Within population graphs, Peng et al. [27] employed a GCN model, utilizing the Pearson correlation matrix of BOLD signals as subject features. It is worth noting that most current GNN models tend to be shallow. However, Cao et al. [32] introduced a 16-layer GCN model designed to extract high-level features effectively. On the other hand, in their subject graph, Ma et al. [96] used the Pearson correlation of the BOLD signal as a node feature and the GCN to extract graph-level features, concatenating them with phenotypic information for prediction. Qin et al. [44] and Gu et al. [45] employed graph theory methods for node feature extraction. Meanwhile, Wagh et al. [81] extracted features from EEG signals in different frequency bands as initial node features.

##### GraphSAGE-Based

In the real-world application scenario, the structure of the graph often undergoes changes. For instance, in the GNN diagnostic model based on a population graph, when a new patient requires diagnosis, that new patient is incorporated into the original population graph, thus altering its structure. Traditional models like GCN struggle to adapt to such graph evolution. To address this issue, the GraphSAGE [78] was introduced and applied in the context of ND diagnosis. Within population graphs, Zheng et al. [34] used the GraphSAGE to partition the graph into mini-batches, avoiding the limitation of calculating on the whole graph and enabling inductive learning on the population graph. Song et al. [98] aggregated node information based on GraphSAGE and modified the activation function. They leveraged risk factors, cognitive test scores, and MRI as features for subject nodes. In subject graphs, Zhu et al. [39] used GraphSAGE for spatial features extraction, while using the Pearson correlation and coordinate position as node features.

##### GAT-Based

Due to the effectiveness of the attention mechanism, researchers have integrated it into GNN, also known as GAT [79]. In the diagnosis of ND, GAT stands out for its ability to adaptively adjust edge weights during the model’s training iterations. Given its prowess in handling weight adaptation, GAT is frequently employed to explore brain connectivity. Safai et al. [100] used GAT to interpret brain connections while extracting structural and functional features from T1-MRI, dMRI, fMRI. Yang et al. [51] and Li et al. [62] used Pearson correlations as node features and GAT to predict ND. Similarly, Yang et al. [47] extracted seven features (number of vertices, surface area, etc.) from sMRI and four features (mean, standard, etc.) from fMRI for each node in the graph. Additionally, Chen et al. [68] incorporated skip connections into GAT.

##### GIN-Based

GIN [80] was proposed to explore the power of the GNN. Presently, most GIN-based diagnostic models for ND operate on subject graphs. Wang et al. [41] used GIN as the main structure of their model and applied feature alignment techniques to mitigate domain shift between the source and target domains. Tao et al. [101] utilized the GIN to concatenate node features from each layer, resulting in the formation of a graph embedding.

##### Others

In addition to the commonly used basic models above, several studies have explored different models. In their population graphs, Rakhimberdina et al. [23] utilized functional connections as node features, while phenotypic features were employed to construct edge weights. They implemented a simple graph convolution method [106], which reduced the computational time of the model. Yang et al. [31] adopted a spectral graph attention network [107] and bilinear aggregator [108] to extract spatial features. Pan et al. [36] employed a multi-scale convolution module based on a snowball GCN [109]. In terms of subject graphs, Wang et al. [40] introduced a GNN model based on Transformer Convolution [110]. Zhao et al. [46] proposed a dynamic graph convolution approach based on EdgeConv [111], enabling the simultaneous aggregation of 1-hop and 2-hop features. Li et al. [61] designed an ROI-aware graph convolutional layer using R-GCN [112] to incorporate both the topological and functional information of the brain network. Mahmood et al. [69] employed a GNN model based on the GRU aggregation function [113].

#### 2.2.2. Spatial-Temporal Feature Extraction

##### RNN-Based

Most RNN-based models [52,59,83,102] employ a sliding window to partition time series data into multiple segments along the time axis, and use graph convolution to extract spatial features, and thus, temporal information is learned through LSTM. For instance, Xing et al. [83] used a sliding window approach to construct their dynamic functional networks. Each functional network served as the graph structure, with the brain ROI volume obtained from T1-MRI used as the node features. These features were input into at each time step of the LSTM. Alternatively, some methods divide the time steps based on the subject’s physical examination schedule. Kim et al. [103] used T1-MRI at multiple time points. They selected GCN as the spatial convolution model and inputted these spatial features into the LSTM to capture temporal information.

##### CNN-Based

Differing from the temporal models based on RNNs, temporal models based on CNNs do not adhere to strict time steps. Yao et al. [104] used sliding windows to divide fMRI into multiple segments. Within each segment, they utilized graph convolution to learn the spatial relationship between ROIs. Subsequently, a CNN was employed to capture the temporal relationships between adjacent segments. Zhdanov et al. [82] used a CNN to extract EEG temporal features, followed by the utilization of a high-order GNN [114] to extract spatial features. Shan et al. [66] introduced a spatial-temporal GNN model, where each spatial-temporal block comprised two temporal convolution layers and one spatial convolution layer. He et al. [71] extracted the trajectory, velocity, and acceleration features from a video of human motion and input them into a two-branch ST-GCN [115] to extract global and local features, respectively.

#### 2.2.3. Multi-Graph Feature Extraction

Graphs derived from different scales or construction methods represent the information from varying perspectives. Consequently, multiple graphs require multiple graph convolution operations to be computed. In the case of multi-scale graphs, Yao et al. [105] used three brain templates to establish multi-scale functional connections. Each template corresponded to a branch of the graph convolution, facilitating the learning of the brain networks at different scales. Similarly, Yao et al. [57] used four templates to create four graphs, each corresponding to a graph convolutional network. For multi-construction graphs, Wu et al. [84] generated three graphs using a phase locking value, phase lag index and Pearson correlation coefficient, respectively. They subsequently utilized spatial-temporal graph convolution to extract EEG features in three branches. In another approach, Yu et al. [56] constructed four graphs based on node features using KNN and percentage thresholding methods. Then, GAT was employed to extract spatial features from these four graphs.

### 2.3. Graph Pooling

Following feature extraction through graph convolution, graph pooling is employed to select the most distinctive and robust features. This process aims to obtain the most informative graph embedding from the node embeddings. While some studies refer to the transition from node embedding to graph embedding as the readout layer or function [53,116,117], there exists no distinct boundary between the graph pooling layer and graph readout layer. Therefore, this review consistently refers to them as graph pooling. Commonly utilized pooling methods include global pooling and hierarchical pooling. A summary of frequently used graph pooling methods is presented in Table 3.

Global pooling methods directly transform node embeddings into graph embeddings. For example, the calculation of summation pooling [28] is shown in Equation (6).

(6) $$H^{f}=\sum_{l}w^{l}⨀H^{l}$$

where, $H^{l}$ is the hidden feature output of each graph convolutional layer, and $w^{l}$ is the adaptive weight of each layer. $H^{f}$ represents the final feature, which can be entered into the fully connected layer and the softmax layer for classification.

Hierarchical pooling gradually reduces the size of the graph layer by layer until the node embeddings ultimately become the graph embeddings. This is one type of hierarchical pooling [61], as depicted in Equation (7). Initially, node features are scored and normalized through vector mapping to obtain $s^{l}$. Then, the top k nodes with the highest scores, as determined by $s^{l}$, are selected. Finally, weights are assigned to the node features, resulting in hidden features with reduced dimensionality.

(7) $$\left\{\begin{array}{c} s^{l}={\tilde{H}}^{l+1}W^{l}/||W^{l}|{|}_{2}\\ {\tilde{s}}^{l}=\frac{s^{l}-\mu \left(s^{l}\right)}{\sigma \left(s^{l}\right)}\\ i=topk\left({\tilde{s}}^{l},k\right)\\ H^{l+1}=({\tilde{H}}^{l+1}⨀sigmoid({\tilde{s}}^{l}){)}_{i,\;:} \end{array}\right.$$

#### 2.3.1. Global Pooling

These methods encompass average pooling, maximum pooling, and summation pooling. Graph average pooling involves calculating the average of node embeddings along a specific dimension to derive a graph embedding. Wagh et al. [81] conducted the average pooling of node embeddings following graph convolution to acquire a graph level representation. Similar approaches are observed in other works, such as [44,55,62,71,99,103].

Graph maximum pooling involves selecting the maximum values from node embeddings along a specific dimension, as demonstrated in works like [65,82]. Additionally, some studies combine maximum pooling with other pooling methods. For example, Lee et al. [38] concatenated the output of summation pooling and maximum pooling to form a graph embedding. Zhao et al. [46] obtained the representation of the whole graph by concatenating the mean and maximum value of node embeddings. Kazi et al. [25] utilized both concatenation and maximum pooling to merge the output of each graph convolution. Subaramya et al. [74] sorted features and extracted significant features with maximum pooling. Mahmood et al. [69] simultaneously used maximum pooling, average pooling, and attention-based pooling [121].

Graph summation pooling is the summing of node embeddings, such as [118]. However, simple graph average and summation pooling may not effectively emphasize crucial node features. Consequently, Kazi et al. [21] employed a weighted summation method based on attention scores to combine each modal feature and generate a representation vector for each subject. Zhang et al. [28] fused the output from each graph convolutional layer using a learnable weighted summation method to produce the final embedding.

#### 2.3.2. Hierarchical Pooling

The aforementioned pooling methods have the potential to introduce noise from less relevant brain regions or overlook the community characteristics of the brain. In contrast, hierarchical pooling progressively reduces the number of nodes layer by layer, which can help eliminate noise disturbance while preserving community attributes. Among the frequently utilized types of hierarchical pooling are TopK pooling [122], SAG pooling [123], and Diff pooling [124].

In studies such as [43,63,119], TopK pooling was used to coarsen the graph. Li et al. [61] used two layers of hierarchical pooling based on TopK pooling, with each reducing the number of nodes by half. The remaining node embeddings take the mean and maximum pooling as the graph-level representation. Likewise, Li et al. [64] utilized TopK pooling and calculated the mean and maximum values of node embeddings to derive a graph representation. Song et al. [37] defined the similarity matrix and calculated the similarity score for each class, and then carried out pooling calculation according to the similarity score and top-K selection.

To solve the problems of isolated nodes and information loss existing in the traditional TopK pooling, Chen at al. [120] proposed a SAG pooling method, performing pooling calculations on both local and global graphs. Ma et al. [96] and Zhang et al. [95] also adopted SAG pooling to reduce the number of nodes in their respective studies.

Given the community properties inherent in brain networks, Yang et al. [47] and Mei et al. [49] employed the Diff pooling method to reduce the number of nodes while preserving subnetworks. Zhu et al. [39] proposed a pooling method including three scales: the global scale, community scale [124], and ROI scale [122]. These scales were utilized to capture the topology of functional networks at multiple levels.

Furthermore, various other graph pooling methods exist. Jiang et al. [30] used Eigen pooling [125] to obtain subgraph features, and then used global average pooling to obtain graph-level features. Kumar et al. [54] followed a similar approach to Jiang et al. [30]. Kong et al. [59] conducted pooling across three scales of brain parcellations.

### 2.4. Graph Prediction

Following feature extraction via graph convolution and feature selection through graph pooling, we obtain node embeddings or graph embeddings. These embeddings serve as the foundation for making predictions at both the node and the whole-graph levels. For node-level predictions, the majority of studies use the population graph for node prediction, because each node on the population graph represents a subject. For graph-level prediction, most studies use the subject graph for graph prediction, since the subject graph extracts features from all brain regions or electrodes to form the representation of subject.

Given the node embedding or graph embedding obtained via graph convolution and graph pooling, we cloud train the GNN model from the perspective of the population graph and subject graph, respectively. Ultimately, this allows us to achieve the goal of graph prediction. Taking the most commonly used cross-entropy loss function as an example, the loss functions of node classification and whole graph classification are shown in Equations (8) and (9), respectively. Y represents the one-hot label. Feature H passes through the fully connected layer and softmax to obtain the final prediction probability Z. C is the number of categories.

(8) $$L_{node}=-\sum_{p\in Y_{L}}\sum_{c=1}^{C}Y_{pc}log(Z_{pc})$$

(9) $$L_{graph}=-\sum_{c=1}^{C}Y_{c}log(Z_{c})$$

In the loss of node classification, $Y_{L}$ is the set of node indexes that have subject labels. In other words, the model is trained in a semi-supervised manner, and the labeled nodes are used to update the model parameters. The whole graph classification loss is a conventional cross-entropy.

In addition to node classification and graph classification, we further divide the types of supervision to include supervised learning, semi-supervised learning, and unsupervised learning. A summary of the graph prediction commonly used in GNN models is shown in Table 4.

#### 2.4.1. Node Classification

Many node classification studies rely on semi-supervised learning, where both the training set and the test set samples are treated as nodes within the graph. During the training phase, only the node labels for the training set are provided, while the labels for the test set remain unknown. For instance, Parisot et al. [24] conducted node feature extraction on the population graph using graph convolution and employed softmax for classification. Cao et al. [32] proposed a deep GNN model to extract advanced node features and introduced a residual structure to avoid gradient vanishing or explosion. They employed cross-entropy to supervise the nodes within the training set. In order to avoid the inconvenience caused by transductive learning on the graph, Song et al. [98] proposed a sampling strategy based on meta-learning. This strategy involved creating a subgraph through sampling from the population graph, effectively transforming semi-supervised learning into supervised learning. Additionally, there are unsupervised learning methods available for node-level classification. These methods leverage unsupervised learning to extract additional information, thereby enhancing the model’s generalization performance. Peng et al. [27] adopted self-supervised learning to extract the features of the fMRI data itself. Wang et al. [29] utilized the contrastive learning method to ensure the features from the same subjects were close to each other, while those from different subjects were distant.

#### 2.4.2. Graph Classification

In the context of graph-level supervised learning, Shan et al. [66] flattened all node features following the convolution calculations. Subsequently, they employed a fully connected layer for classification. Lee et al. [38] used an end-to-end approach to optimize the network; a supervised learning-optimized temporal embedding network, regional relation representation network, and classifier. And reinforcement learning optimized the ROI selection network. Finally, individual networks were classified. Zhu et al. [117] used contrastive learning to combine structural and functional information to form a graph-level embedding, and employed both cross-entropy and contrastive loss to jointly optimize the model. Li et al. [62] utilized an MLP as a classifier, combined with cross-entropy loss, distance loss, and group-level consistency loss, to classify the subject graph. Yao et al. [57] implemented a mutual learning strategy based on KL divergence to fuse four graph convolution branches. For semi-supervised and unsupervised learning, Kong et al. [50] made use of prior information from labeled samples through semi-supervised learning. Wang et al. [41] proposed a domain-adaptive approach based on the feature alignment strategy for ND classification. Zhao et al. [43] pre-trained an encoder via self-supervision, and subsequently conducted ND classification through an MLP.

#### 2.4.3. Explainability and Interpretability

The model’s explainability and interpretability play a crucial role in extracting biomarkers and investigating important brain regions and connections in the brain. GNN-based ND diagnosis primarily leverages the attention mechanism, class activation mapping (CAM) [126,127], and pooling score.

For the attention mechanism, Zhang et al. [95] utilized fMRI data for classifying subjective cognitive decline via the GCN model. They employed the attention mechanism to identify important brain regions. Wang et al. [40] designed a graph convolution model based on the attention mechanism for ND diagnosis and the extraction of image biomarkers. Additionally, they conducted an analysis of the correlation between image biomarkers and genes. Zhang et al. [28] proposed the local-to-global GNN. They modeled a local graph based on individual-level functional connection and a global graph based on population-level non-image information to capture both local and global features. Significant brain regions were extracted through self-attention scores.

In the context of CAM methods, Lei et al. [128] employed a GNN model for ND diagnosis and identified salient brain regions using CAM. They also used ComBat [129] to mitigate cross-site effects. Qin et al. [55] validated the classification results of a graph convolution model using large-scale and multi-site data. They extracted significant brain regions in conjunction with CAM and calculated metrics such as degree, betweenness, and efficiency for these salient brain regions. Zhou et al. [92] proposed an interpretable method based on GradCAM [130] to find salient brain regions and classify NDs through a GCN model combined with multi-modal data.

During the graph dimensionality reduction process, pooling scores serve as indicators of node importance, and some studies employ these scores as biomarkers. For instance, Li et al. [61] proposed the BrainGNN model, incorporating ROI-aware graph convolutional layers and the ROI-selection pooling layers. They made modifications to TopK pooling and used the projection of node embeddings as the scores of salient brain regions. Zhu et al. [39] proposed a GNN model based on triple pooling, aimed at learning multi-scale topologies within functional networks. They employed various pooling methods to extract significant brain regions as biomarkers.

Other explainability and interpretability methods used shared weights [131] and reinforcement learning [38]. Cui et al. [131] proposed an interpretable GNN model called IBGNN, which achieved the extraction of significant brain regions and important connections at the group level through weight sharing. Additionally, Cui et al. [118] proposed the BrainNNExplainer model, building upon the BrainNN [117] framework, and employed a shared mask as an interpretation generator to highlight the meaningful connectivity within disease-specific brain networks. Lee et al. [38] combined reinforcement learning with a GNN to select individualized important nodes. Gu et al. [45] utilized a GCN to assess the impact of node removal on experimental results, aiding in the identification of important nodes.

## 3. Graph Neural Network Application in ND Diagnosis

In this section, we broadly investigated common NDs diagnosed using a GNN. At the same time, we investigated the data modality, number of subjects, and diagnostic accuracy, etc. A summary of the GNN diagnosis of NDs as shown in Table 5. We provided more details in the Appendix A. The diagnostic information of AD, PD, ASD, SZ, MDD, BP, EP and ADHD can be obtained in Table A1, Table A2, Table A3, Table A4, Table A5, Table A6, Table A7 and Table A8 respectively. 

**Figure 4:**
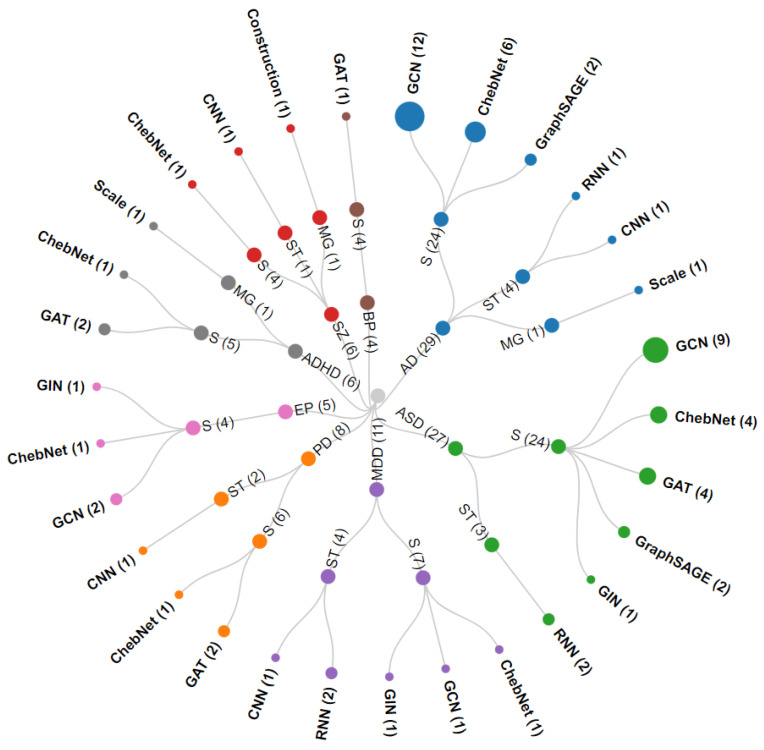
Feature extraction of ND diagnosis. S represents spatial features, T denotes temporal features, and MG represents multi-graph features. The outermost ring represents the model for feature extraction. (:) denotes the number of studies. This circular dendrogram was drawn from the works listed in Table 5.

**Figure 5:**
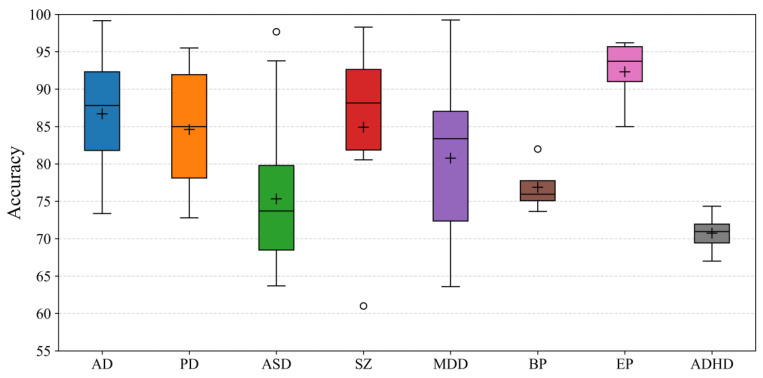
The accuracy of the ND diagnosis. Plus markers represent the mean value of accuracy, and circles represent outliers. As can be seen from the figure, the mean accuracy of AD, PD, ASD, SZ, MDD, BP, EP, and ADHD diagnosis is about 87%, 85%, 75%, 85%, 81%, 77%, 92%, and 71%, respectively. This boxplot was drawn from the works listed in Table 5.

The feature extraction of the ND diagnosis is shown in Figure 4. We can observe from the figure that most research is on extracting spatial features, while the research on extracting multi-graph features is less prevalent. For the models of feature extraction, ChebNet and GCN are the most researched, which were the first proposed. The accuracy of the ND diagnosis is shown in Figure 5. As can be seen from the figure, the mean accuracy of AD, PD, ASD, SZ, MDD, BP, EP, and ADHD diagnosis is about 87%, 85%, 75%, 85%, 81%, 77%, 92%, and 71%, respectively.

### 3.1. Alzheimer’s Disease

Alzheimer’s disease (AD) is an irreversible neurodegenerative disease that destroys memory and cognition [160]. A GNN can be used to classify subjects into healthy control (HC), mild cognitive impairment (MCI), and AD.

In the studies of diagnosis using unimodal data, Mei et al. [49] proposed a hierarchical GNN model for MCI diagnosis based on fMRI. They implemented limited messaging across different hierarchical levels to prevent over-smoothing and employed clustering-based hierarchical pooling to extract graph representations. Wang et al. [137] sampled fMRI in adjacent spaces and adjacent times to learn the spatial-temporal features. In addition to using MRI data, Klepl et al. [65] used EEG data to classify AD patients. They employed eight functional connectivity measures to estimate the brain graph. And in work [66], the spatial-temporal GCN could jointly learn cross-channel topological information and channel-specific temporal information.

In the studies of diagnosis using multimodal data, Choi et al. [139] proposed an adaptive scale aggregation of adjacent node features to diagnose AD based on dMRI and PET. More studies combined image and non-image data, Xing et al. [83] took demographic information prediction as the auxiliary task and used T1-MRI and fMRI to predict MCI. Jiang et al. [30] developed a hierarchical GCN model that combined individual brain networks and global population networks to better learn graph embedding. Kazi et al. [25] presented the InceptionGCN model based on multi-kernel graph convolution for AD classification. This multi-kernel graph convolution approach was designed to capture graph structural heterogeneity. Liu et al. [26] extracted features such as gray matter volume and shortest path length from subjects using T1-MRI and fMRI, and they employed a multi-task selection method to obtain effective features for MCI diagnosis. Song et al. [37] integrated fMRI and dMRI through a multi-center and multi-channel pooling for early AD diagnosis. Zheng et al. [34] proposed a multi-modal graph learning framework that incorporated a modality-aware representation learning module to extract multi-modal correlation and complementary information. Yang et al. [31] introduced a multimodal adaptive fusion graph network, consisting of a spectral graph attention module, bilinear aggregation module, and adaptive fusion module.

Other studies have focused on predicting the conversion of MCI to AD [24,28,33,34,98,103,136]. Wee et al. [136] employed the Chebyshev graph convolution to predict MCI conversion outcomes. Huang et al. [33] constructed a population graph based on MRI, PET, and non-image information and made predictions. Song et al. [98] used meta-learning to address the challenge of inductive learning on the population graph. They achieved this by constructing subgraph and aggregation node information, effectively transferring known node information to the nodes being predicted. Kim et al. [103] proposed a temporal GNN model for the prognosis of MCI and utilized GNNExplainer [161] to extract important brain regions.

### 3.2. Parkinson’s Disease

Parkinson’s disease (PD) is a neurodegenerative disease that presents with motor and non-motor symptoms, including tremor, sleep disturbances, and dementia [162].

In the studies involving diagnosis using unimodal data, Huang et al. [72] proposed a multi-task graph representation learning framework based on node clustering. The model not only diagnosed early PD, but also output clinical scores. In addition to using medical imaging data, He et al. [71] introduced an asymmetric dual-branch spatiotemporal graph convolutional network. This network was designed to learn global and local information from a human skeleton video to predict PD.

In the studies focusing on multimodal data, Zhang et al. [99] proposed a classification model that facilitated cross-modal learning between structural and functional networks for PD diagnosis. The loss function employed not only cross-entropy, but also the local and global decoding loss of edge reconstruction. Safai et al. [100] extracted multimodal features from T1-MRI, dMRI, and fMRI, and used GAT to diagnose PD. Kazi et al. [21] used non-image data to construct multiple graphs. The GCN model was then employed to learn the topological relationship within each graph. Additionally, they utilized an LSTM-based attention mechanism to fuse multimodal information.

### 3.3. Autism Spectrum Disorder

Autism spectrum disorder (ASD) is a neurodevelopmental disorder characterized by social communication deficits and repetitive behaviors [163].

In the studies focusing on the use of unimodal data for diagnosis, fMRI is the most commonly employed modality. Ktena et al. [89] introduced metric learning within a Siamese GCN to learn the graph similarity. They also introduced a constrained variance loss function to enhance the model’s ability to predict ASD. Li et al. [61] proposed the BrainGNN model in which they designed the ROI-aware graph convolutional layers and the ROI-selection pooling layers. To enhance ROI selection and align individual-level patterns with group-level patterns, they proposed three regularization terms: unit loss, TopK pooling loss, and group-level consistency loss. Noman et al. [102] proposed a graph autoencoder to learn the dynamic brain network. Cao et al. [52] developed a graph structure-aware model for learning the dynamic brain network. They split fMRI into multiple segments using a sliding window and coarsened the graph through graph clustering. Cao et al. [48] proposed a three-stage GNN-based framework for ASD diagnosis. The framework included graph structure learning, graph generation learning, and graph embedding learning.

In the studies of diagnosis using multimodal data, Chen et al. [68] introduced a graph attention neural network that leveraged adversarial learning, utilizing both T1-MRI and fMRI. Lin et al. [35] constructed a robust population graph and employed a message-passing approach to eliminate noise and adapt to heterogeneous data from multiple sites. Cao et al. [32] proposed a 16-layer GCN model for the extraction of high-level features. In order to avoid gradient vanishing, over-fitting, and over-smoothing, they integrated ResNet [164] and DropEdge [165] strategies into the model.

### 3.4. Schizophrenia

Schizophrenia (SZ) is a neurodevelopmental disorder characterized by paranoid delusions and auditory hallucinations [166].

In studies involving diagnosis using unimodal data, Yu et al. [56] introduced a multigraph attention graph convolutional network and bilinear convolution network, and used fMRI to diagnose SZ. Mahmood et al. [69] employed multi-head self-attention to learn functional connections. Zhdanov et al. [82] proposed a spatial-temporal graph convolution model based on a high-order GNN [114]. The GNNExplainer [161] was used to calculate the importance score for each node, each edge, and each time point.

In the studies on diagnosis using multimodal data, Chang et al. [63] predicted first-episode SZ, chronic SZ, and HC based on EEG and demographic information. Yang et al. [67] used GRU to extract node features from functional and structural networks. They constructed an adjacency matrix based on the inner product of these node features and applied bilateral graph convolution for the diagnosis of SZ.

### 3.5. Major Depressive Disorder

Major depressive disorder (MDD) is characterized by sadness or irritability, accompanied by psychophysiological changes such as sleep disturbance, loss of ability to enjoy life at work and with friends, crying, and suicidal thoughts [167].

The diagnosis of MDD mainly uses EEG and fMRI; Kong et al. [59] proposed a spatiotemporal graph convolutional network for MMD diagnosis. They constructed a dynamic functional connection matrix using a sliding window, applied spatial graph attention convolution to learn important brain regions, and obtained the graph representation through hierarchical pooling. Finally, the temporal fusion module learned the dependence of multiple time steps based on fMRI. Wang et al. [58] employed the topological features of brain regions through an attention-enhanced graph convolutional network based on Transformer [168]. Kong et al. [50] proposed a multi-stage graph fusion model based on the functional connectivity between gray matter and white matter. In studies involving EEG data, Chen et al. [120] proposed a self-attention graph pooling model, with the loss function incorporating both the clinical scale and ground-truth as the supervision item.

In addition, there are multimodal studies; Pan et al. [36] proposed a comprehensive GNN model that combines functional image features and phenotypic features for MDD diagnosis. Chen et al. [152] presented a modal-shared modal-specific GNN, which aimed to capture the heterogeneity or homogeneity within multimodal data and explore potential relationships between subjects. The model was verified using EEG and audio data.

### 3.6. Bipolar Disorder

Bipolar disorder (BP) is a recurrent chronic disorder characterized by mood and energy fluctuations. It leads to cognitive and functional impairments and increases mortality, especially by suicide [169].

Yang et al. [47] combined T1-MRI and fMRI to classify BP through a cerebral cortex analysis method based on GAT. Zhu et al. [117] proposed a BrainNN method that fused fMRI and dMRI using contrastive learning and aggregated node features via MLP. To learn the potential correlation information of their multi-view graph, Zhao et al. [170] introduced a multi-view graph representation learning framework. Within this framework, a bridge module utilized a tensor decomposition algorithm to extract latent correlation information from multiple views.

### 3.7. Epilepsy

Epilepsy (EP) is one of the most common brain conditions, characterized by a disturbance of electrical activity, as well as repeated and unpredictable seizures [171].

Most epilepsy diagnosis studies use EEG data. Li et al. [158] proposed a structure-generated GNN model for learning the spatial-temporal dynamic features of EEG signals. Tao et al. [101] constructed dynamic brain networks from EEG and used a GIN model to predict seizure. Zeng et al. [94] presented a hierarchy GNN combined with tree classification for epileptic detection. In addition, Dissanayake et al. [172] utilized the individualized graph to predict seizures one hour before they happened based on the CHB-MIT [155] and Siena EEG [173] datasets.

### 3.8. Attention Deficit Hyperactivity Disorder

Attention-deficit/hyperactivity disorder (ADHD) is a heterogeneous and multifactorial disorder characterized by behavioral symptoms of inattention, hyperactivity, and impulsivity [174].

In studies using fMRI data, Ji et al. [53] proposed a hypergraph attention network to learn higher-order structural information and diagnose ADHD. Yao et al. [57] introduced a multi-scale graph convolution model, which used triplet loss to learn similarities among subjects and mutual learning strategies to capture the complementary information of different scale graphs. Zhao et al. [46] proposed a dynamic GNN that simultaneously aggregated the features of first-order and second-order neighborhood nodes. In studies involving multimodal data, Rakhimberdina et al. [23] leveraged phenotypic information and fMRI data to construct a population graph and employed a simple graph convolution model for ADHD diagnosis. Yao et al. [60] applied a heterogeneous graph network to diagnose ADHD.

## 4. Challenges and Outlook

In this section, we summarize the current research challenges and future research directions for GNN models, including graph representation, individual heterogeneity, small sample sizes, domain generalization, and multimodality.

### 4.1. Graph Representation

The graph representation affects the feature extraction of GNN models. Each graph representation method [175,176,177,178] has its own advantages and disadvantages. Recently, predefined methods based on prior knowledge have been widely used, but the classification results were influenced by different datasets. In addition, predefined methods may be affected by factors unrelated to the diagnostic prediction task, such as gender. Adaptive methods may be suitable alternatives because they can optimize the graph through the iteration of the model in the training process and reduce the workload of hyperparameter tuning.

### 4.2. Individual Heterogeneity

Each subject’s brain has individual heterogeneity. Suppressing individual heterogeneity can reveal commonalities of diseases, which further helps researchers and physicians understand the mechanisms involved and diagnose diseases [179,180]. There are two directions which may be useful to suppress individual heterogeneity in GNN models:

(1) Node constraint. Projection methods can be used to obtain the weight of the node, and the weight can be constrained by the group-level consistency loss, so that the weight distribution in the same group tends to be consistent [61].
(2) Edge constraint. The intra-group similarity and inter-group difference in functional connections can be reduced by adding variance loss and 2-norm loss [181].

### 4.3. Small Sample Sizes

Compared with computer vision and natural language processing, medical data collection is more resource-intensive, so the amount of medical data is often small. The traditional method to solve above problem is to augment the data. However, it is not enough to solve the over-fit problem of the GNN models [61]. Therefore, combining data augmentation with self-supervised learning may be a direction to pursue [27,29,43,88,138].

Self-supervised learning can use the information contained in the data itself to improve the performance of the model. For example, the GNN models can be pretrained using the self-supervised loss function, and then be fine-tuned and used for downstream tasks.

### 4.4. Domain Generalization

Domain generalization is affected by different acquisition protocols, imaging equipment, imaging parameters, inclusion criteria, and other factors; the data collected by different centers often have distribution bias. This results in the generalization problem of GNN models. Domain generalization and domain adaptation, as two kinds of transfer learning, may be future research directions for GNN model optimization [41,54,136,140,182]. For instance, domain adaptation can be used to train GNN models on cross-site and cross-disease datasets.

### 4.5. Multimodality

With the popularization and upgrading of neuroimaging equipment, it is possible for patients to perform multiple imaging examinations at the same time. Different images can reflect different pathological information. T1-MRI studies the brain morphologically. fMRI reflects the spatial and temporal associations of the brain. DTI reflects white matter fiber bundle connections. Multimodal images together provide complementary information, which can depict the patient’s state more comprehensively. However, it is a challenge to combine multimodal information using GNN models [83,92,139]. The idea of multiple graphs may be a research direction to pursue. The multiple graph techniques can filter out redundant information and fuse the information from different modes effectively.

## 5. Conclusions

It is of great importance to diagnose NDs by combining GNN technology and brain imaging. In this study, we provided an overview and outlook on GNN applications in the diagnosis of ND. Firstly, different modules of GNNs, including graph construction, graph convolution, graph pooling, and graph prediction were systematically introduced; secondly, we compared different GNN applications in terms of data modality, number of subjects, and diagnostic accuracy; finally, we discussed challenges in GNNs, including optimizations for graph representation, individual heterogeneity, small sample sizes, domain generalization, and multimodality. The results of this review may be a valuable contribution to the ongoing intersection of artificial intelligence technology and brain imaging.

## Figures and Tables

**Table 1:** A summary of similarity and dissimilarity methods commonly used in graph construction.

Form	Methods	Works
Population Graph	Hamming Distance	[23]
	Correlation Distance	[21,24,25,26,27,28]
	Euclidean Distance	[29,30,31],
	Pearson Correlation	[32]
	Cosine Similarity	[33,34,35,36],
	Attention Mechanism	[37]
Subject Graph	Correlation Distance	[38]
	Pearson Correlation	[39,40,41,42,43,44,45,46,47,48,49,50,51,52,53,54,55,56,57,58,59,60]
	Partial Correlation	[61,62,63,64],
	Mutual Information	[65]
	Phase Lag Index	[63,66]
	Inner Product	[67]
	Attention Mechanism	[68,69]

**Table 2:** A summary of graph convolutions commonly used in GNN models.

Feature Extraction	Convolution	Works
Spatial	ChebNet-based	[21,24,26,30,33,55,63,73,88,89,90]
	GCN-based	[27,32,35,37,38,44,45,48,49,50,54,74,81,91,92,93,94,95,96,97]
	GraphSAGE-based	[34,39,98]
	GAT-based	[47,51,53,60,62,68,99,100]
	GIN-based	[41,43,64,101]
Spatial-Temporal	RNN-based	[52,59,83,102,103]
	CNN-based	[66,71,82,104]
Multi-Graph	Scale	[57,105]
	Construction	[56,84]

**Table 3:** A summary of graph pooling commonly used in GNN models.

Pooling	Methods	Works
Global Pooling	Average Pooling	[44,55,62,71,81,99,103]
	Maximum Pooling	[25,38,46,65,69,74,82]
	Summation Pooling	[21,28,118]
Hierarchical Pooling	TopK Pooling	[37,43,61,63,64,119]
	SAG Pooling	[95,96,120]
	Diff Pooling	[39,47,49]
	Eigen Pooling	[30,54]

**Table 4:** A summary of graph prediction level in GNN models.

Prediction Level	Supervision Type	Works
Node Classification	Supervised Learning	[98]
	Semi-supervised Learning	[24,26,32,33,34,36]
	Unsupervised Learning	[27,29,88]
Graph Classification	Supervised Learning	[38,45,48,52,73,89]
	Semi-supervised Learning	[50]
	Unsupervised Learning	[41,43]

**Table 5:** Summary of GNN diagnosis of NDs.

Disease	Dataset	Modality	Number of Subjects	ACC	Works
AD	ADNI [132], OASIS [133], TADPOLE [134], Blackburn et al. [135], In-house	EEG	39–40	91.1–92.0%	[65,66]
		T1-MRI	2442	85.8%	[136]
		dMRI	162–367	86.0–97%	[57,74]
		fMRI	91–1326	73.37–99.16%	[38,44,45,49,54,137,138]
		Multimodal	114–1615	75.6–96.0%	[21,25,26,28,30,31,33,34,37,67,83,91,92,98,139,140,141]
PD	PPMI [142], Xuanwu [143], Parkinson Speech [144], In-house	dMRI	194–754	79.82–95.5%	[72,131]
		Video	191	84.1%	[71]
		Multimodal	68–324	72.8–94.6%	[21,29,67,99,100]
ASD	Biopoint Autism Study Dataset [145], ABIDE [146], In-house	EEG	96	93.78%	[93]
		fMRI	118–1112	66.03–79.8%	[39,40,48,51,52,53,58,61,62,64,102]
		Multimodal	866–1029	63.7–89.77%	[23,24,25,27,28,30,32,33,34,35,36,68,91,96,140]
SZ	COBRE ^1^, CHUV [147], In-house	EEG	81	61%	[82]
		fMRI	125–1412	85.8–90.48%	[56,128]
		Multimodal	54–145	80.6–98.3%	[23,63,67]
MDD	MODMA [148], REST-meta-MDD [149], DAIC-WOZ [150], In-house	EEG	53	84.91%	[120]
		fMRI	84–2361	63.6–93%	[42,43,50,55,58,59,104,151]
		Multimodal	226–533	89.13–99.24%	[36,152]
BP	Cao et al. [153], In-house	fMRI	97	75.56%	[118]
		dMRI	97	76.33%	[131]
		Multimodal	97–106	73.64–82%	[47,117]
EP	TUH [154], CHB-MIT [155], Max Planck Institute Leipzig Mind-Brain-Body [156], Freiburg iEEG [157]	EEG	9–6746	85–96.2%	[81,90,94,101,158]
ADHD	ADHD-200 [159], In-house	fMRI	520–627	67.00–72.0%	[46,53,57,88]
		Multimodal	187–714	70.1–74.35%	[23,60]

^1^ COBRE: The Center for Biomedical Research Excellence, http://fcon_1000.projects.nitrc.org/indi/retro/cobre.html (accessed on 1 September 2023).

## Data Availability

Data availability is not applicable to this article as no new data were created or analyzed in this study.

## Appendix A

The details of the diagnosis of ND based on GNNs are shown in Table A1, Table A2, Table A3, Table A4, Table A5, Table A6, Table A7 and Table A8. In the ‘Feature’ column of these tables, S represents spatial features, T denotes temporal features, and MG represents multi-graph features. The ‘-’ is followed by the model’s name. Column ‘ACC’ represents accuracy with mean (standard deviation). ‘MMSE’ denotes Mini-Mental State Examination, ‘FDG-PET’ denotes 18F-fluorodeoxyglucose PET, ‘AV45-PET’ denotes 18F-florbetapir PET, ‘Amyloid-PET’ denotes amyloid PET, ‘ApoE’ denotes apolipoprotein E, and ‘CSF’ denotes cerebro-spinal fluid.

**Table A1:** Diagnosis of AD.

Authors	Modality	Dataset	Number of Subjects	ACC (%)	Feature
Klepl et al. [65]	EEG	Blackburn et al. [135]	40	91.9 (0.4)	S-Other
Shan et al. [66]	EEG	In-house	39	91.1	ST-CNN
Wee et al. [136]	T1-MRI	ADNI, In-house	2442	85.8 (0.8)	S-ChebNet
Subaramya et al. [74]	dMRI	ADNI	162	97.0	S-GCN
Yao et al. [57]	dMRI	ADNI	367	86.0 (1.3)	MG-Scale
Gu et al. [45]	fMRI	ADNI	311	94.7	S-GCN
Lee et al. [38]	fMRI	ADNI	101	74.4 (1.8)	S-GCN
Qin et al. [44]	fMRI	ADNI	91	83.3	S-GCN
Kumar et al. [54]	fMRI	ADNI	189	81.8	S-GCN
Wang et al. [137]	fMRI	OASIS	1000	99.1	ST-Other
Tang et al. [138]	fMRI	OASIS	1326	77.5 (1.8)	S-GCN
Mei et al. [49]	fMRI	ADNI	483	73.3	S-GCN
Liu et al. [26]	T1-MRI, fMRI, gender, age, MMSE	ADNI	210	84.1	S-ChebNet
Xing et al. [83]	T1-MRI, fMRI, demographic information	ADNI	368	79.7	ST-RNN
Zhou et al. [92]	T1-MRI, FDG-PET, AV45-PET	ADNI	755	81.8 (3.1)	S-GCN
Song et al. [37]	fMRI, DTI, gender, device information, site	ADNI, In-house	459	95.7	S-GCN
Song et al. [98]	age, gender, ApoE, T1-MRI, etc.	TADPOLE	1615	94.4	S-GraphSAGE
Choi et al. [139]	DTI, Amyloid-PET, FDG-PET	ADNI	401	96.0 (2.8)	S-Other
Yang et al. [31]	T1-MRI, gender, etc.	TADPOLE	557	92.8	S-Other
Huang et al. [33]	Phenotypic data, MRI, ApoE, FDG-PET, etc.	TADPOLE	557	87.8	S-ChebNet
Zheng et al. [34]	MRI, PET, cognitive tests, CSF, risk factors, demographic information	TADPOLE	603	92.3 (1.7)	S-GraphSAGE
Kazi et al. [25]	PET, CSF, etc.	TADPOLE	557	88.5 (3.3)	S-ChebNet
Zhang et al. [28]	fMRI, age, gender, site	ADNI	134	82.1 (1.4)	S-GCN
Peng et al. [91]	fMRI, T1-MRI, age, etc.	ADNI	911	75.8 (0.7)	S-GCN
Jiang et al. [30]	fMRI, age, gender, site	ADNI	133	75.6 (0.2)	S-ChebNet
Li et al. [140]	fMRI, gender, etc.	ADNI	133	89.4 (0.4)	S-GCN
Kazi et al. [21]	PET, CSF, etc.	TADPOLE	564	83.3 (3.9)	S-ChebNet
Yang et al. [67]	fMRI, DTI	ADNI	114	90.4 (2.4)	S-Other
Zhu et al. [141]	fMRI, age, etc.	ADNI	291	88.18	ST-Other

**Table A2:** Diagnosis of PD.

Authors	Modality	Dataset	Number of Subjects	ACC (%)	Feature
Huang et al. [72]	DTI	PPMI	194	95.5	S-Other
Cui et al. [131]	DTI	PPMI	754	79.8 (1.4)	S-Other
He et al. [71]	Video	In-house	191	84.1	ST-CNN
Zhang et al. [99]	fMRI, dMRI	PPMI	323	72.8	S-GAT
Kazi et al. [21]	T1-MRI, demographic information, etc.	PPMI	324	91.0 (4.6)	S-ChebNet
Safai et al. [100]	T1-MRI, dMRI, fMRI	In-house	109	73.0	S-GAT
Yang et al. [67]	fMRI, DTI	Xuanwu [143]	155	85.9 (4.5)	S-Other
Zhang et al. [29]	voice, gender, etc.	Parkinson Speech, PPMI	68	94.6 (1.4)	ST-Other

**Table A3:** Diagnosis of ASD.

Authors	Modality	Dataset	Number of Subjects	ACC (%)	Feature
Wadhera et al. [93]	EEG	In-house	96	93.7	S-GCN
Li et al. [61]	fMRI	Biopoint Autism Study Dataset	118	79.8 (3.6)	S-Other
Li et al. [64]	fMRI	Biopoint Autism Study Dataset	118	76.0 (6.0)	S-GIN
Li et al. [62]	fMRI	Biopoint Autism Study Dataset	118	79.7 (5.1)	S-GAT
Cao et al. [48]	fMRI	ABIDE	1112	72.8 (0.8)	S-GCN
Zhu et al. [39]	fMRI	ABIDE	1112	72.4 (3.6)	S-GraphSAGE
Yang et al. [51]	fMRI	ABIDE	871	67.2	S-GAT
Wang et al. [40]	fMRI	ABIDE	884	79.7	S-Other
Noman et al. [102]	fMRI	ABIDE	144	66.0 (7.1)	ST-RNN
Wang et al. [58]	fMRI	ABIDE	629	66.9 (0.9)	ST-Other
Ji et al. [53]	fMRI	ABIDE	1096	70.9	S-GAT
Cao et al. [52]	fMRI	ABIDE	871	68.4	ST-RNN
Ma et al. [96]	fMRI, phenotypic information	ABIDE	988	78.10	S-GCN
Zheng et al. [34]	fMRI, phenotypic information	ABIDE	871	89.7 (2.7)	S-GraphSAGE
Kazi et al. [25]	fMRI, phenotypic information, etc.	ABIDE	871	69.2 (6.6)	S-ChebNet
Peng et al. [27]	fMRI, phenotypic information	ABIDE	1029	63.7 (1.8)	S-GCN
Chen et al. [68]	T1-MRI, fMRI	ABIDE	1007	74.7	S-GAT
Zhang et al. [28]	fMRI, age, gender, site	ABIDE	871	81.7 (1.1)	S-GCN
Peng et al. [91]	fMRI, T1-MRI, age, etc.	ABIDE	1029	66.7 (0.6)	S-GCN
Jiang et al. [30]	fMRI, age, gender, site	ABIDE	866	67.2 (0.3)	S-ChebNet
Li et al. [140]	fMRI, gender, etc.	ABIDE	871	76.5 (0.3)	S-GCN
Cao et al. [32]	fMRI, gender, etc.	ABIDE	871	73.7	S-GCN
Huang et al. [33]	Phenotypic information, T1-MRI, ApoE, FDG-PET, etc.	ABIDE	871	81.0 (4.8)	S-ChebNet
Parisot et al. [24]	fMRI, T1-MRI, site, gender, age, etc.	ABIDE	871	70.4	S-ChebNet
Rakhimberdina et al. [23]	fMRI, non-image	ABIDE	871	68.5 (4.3)	S-Other
Pan et al. [36]	fMRI, site, gender, etc.	ABIDE	871	97.6	S-Other
Lin et al. [35]	fMRI, gender, etc.	ABIDE	871	80.7	S-GCN

**Table A4:** Diagnosis of SZ.

Authors	Modality	Dataset	Number of Subjects	ACC (%)	Feature
Zhdanov et al. [82]	EEG	In-house	81	61.0 (1.5)	ST-CNN
Yu et al. [56]	fMRI	COBRE	125	90.4 (1.4)	MG-Construction
Lei et al. [128]	fMRI	In-house	1412	85.8	S-Other
Rakhimberdina et al. [23]	fMRI, non-image	COBRE	145	80.5 (10.8)	S-Other
Chang et al. [63]	EEG, demographic information	In-house	120	93.3	S-ChebNet
Yang et al. [67]	fMRI, DTI	CHUV	54	98.3 (5.0)	S-Other

**Table A5:** Diagnosis of MDD.

Authors	Modality	Dataset	Number of Subjects	ACC (%)	Feature
Chen et al. [120]	EEG	MODMA	53	84.9	S-Other
Yao et al. [104]	fMRI	REST-meta-MDD	533	73.8 (4.8)	ST-CNN
Kong et al. [59]	fMRI	In-house	277	84.1	ST-RNN
Qin et al. [55]	fMRI	In-house	1586	81.5	S-ChebNet
Wang et al. [58]	fMRI	In-house	145	83.2 (1.2)	ST-Other
Pitsik et al. [42]	fMRI	In-house	84	93.0	S-Other
Wang et al. [151]	fMRI	REST-meta-MDD	533	63.6	S-Other
Zhao et al. [43]	fMRI	REST-meta-MDD	2361	64.8	S-GIN
Kong et al. [50]	fMRI	In-house	218	70.9	S-GCN
Pan et al. [36]	fMRI, site, gender, etc.	REST-meta-MDD	533	99.2	S-Other
Chen et al. [152]	EEG, audio	DAIC-WOZ, MODMA	226	89.1	ST-RNN

**Table A6:** Diagnosis of BP.

Authors	Modality	Dataset	Number of Subjects	ACC (%)	Feature
Cui et al. [118]	fMRI	Cao et al. [153]	97	75.5	S-Other
Cui et al. [131]	DTI	Cao et al. [153]	97	76.3 (13.0)	S-Other
Yang et al. [47]	T1-MRI, fMRI	In-house	106	82.0 (3.8)	S-GAT
Zhu et al. [117]	fMRI, DTI	Cao et al. [153]	97	73.6	S-Other

**Table A7:** Diagnosis of EP.

Authors	Modality	Dataset	Number of Subjects	ACC (%)	Feature
Li et al. [158]	EEG	TUH	307	91.0	ST-Other
Tao et al. [101]	EEG	CHB-MIT	22	96.2	S-GIN
Wagh et al. [81]	EEG	TUH, Max Planck Institute Leipzig Mind-Brain-Body	1593	85.0 (4.0)	S-GCN
Lian et al. [90]	EEG	Freiburg iEEG	9	95.6 (0.3)	S-ChebNet
Zeng et al. [94]	EEG	CHB-MIT, TUH	6746	93.7	S-GCN

**Table A8:** Diagnosis of ADHD.

Authors	Modality	Dataset	Number of Subjects	ACC (%)	Feature
Zhao et al. [46]	fMRI	ADHD-200	603	72.0 (1.8)	S-Other
Yao et al. [57]	fMRI	ADHD-200	627	71.8 (1.5)	MG-Scale
Ji et al. [53]	fMRI	ADHD-200	520	69.2	S-GAT
Wang et al. [88]	fMRI	ADHD-200	596	67.0 (3.7)	S-ChebNet
Yao et al. [60]	fMRI, dMRI	In-house	187	70.1 (3.5)	S-GAT
Rakhimberdina et al. [23]	fMRI, non-image	ADHD-200	714	74.3 (4.7)	S-Other
